# Virtual Reality-Based Dichoptic Therapy in Acquired Brain Injury: Functional and Symptom Outcomes

**DOI:** 10.3390/jcm15031004

**Published:** 2026-01-27

**Authors:** Carla Otero-Currás, Francisco J. Povedano-Montero, Ricardo Bernárdez-Vilaboa, Pilar Rojas, Rut González-Jiménez, Gema Martínez-Florentín, Juan E. Cedrún-Sánchez

**Affiliations:** 1 Optometry and Vision Department, Faculty of Optics and Optometry, Complutense University of Madrid, 28037 Madrid, Spain; carlaote@ucm.es (C.O.-C.); ricardob@ucm.es (R.B.-V.); rutgon03@ucm.es (R.G.-J.); gemartin@ucm.es (G.M.-F.); jcedrun@ucm.es (J.E.C.-S.); 2 Hospital Doce de Octubre Research Institute (i+12), 28041 Madrid, Spain; 3 Ramon Castroviejo Institute for Ophthalmic Research, Complutense University of Madrid, 28037 Madrid, Spain; pilar.rojas.lozano@gmail.com; 4 Madrid Eye Institute, Gregorio Marañón General University Hospital, 28007 Madrid, Spain; 5 Applied Vision Research Group, Faculty of Optics and Optometry, Universidad Complutense de Madrid, 28037 Madrid, Spain

**Keywords:** acquired brain injury, dichoptic therapy, virtual reality, binocular vision, visual field, oculomotor rehabilitation

## Abstract

**Background**: Acquired brain injury (ABI) often disrupts binocular vision, causing deviations on the cover test and reduced stereopsis that impair functional visual performance. This study investigated the effects of a dichoptic vision therapy protocol—based on an immersive virtual reality (VR) system—on visual field parameters, oculomotor reaction times, and self-reported visual symptoms in adults with ABI. **Methods**: In a controlled parallel-group design, adult ABI patients (median age 51 years) were assigned to an experimental group (dichoptic VR therapy) or a control group. Six sessions of visual therapy were performed. Primary outcomes included perimetric visual field indices and oculomotor reaction times; the secondary outcome was the Brain Injury Vision Symptom Survey (BIVSS) score. Etiology (stroke vs. traumatic brain injury) was recorded. **Results**: No statistically significant improvements were found in perimetric visual field indices (*p* > 0.05), except for a slight gain in the top-right quadrant in the experimental group. Reaction times did not differ significantly between groups. However, the experimental group reported a greater reduction in visual symptoms as measured by the BIVSS. Patients with traumatic brain injury exhibited better functional improvement, particularly in the top-left quadrant (*p* = 0.04). **Conclusions**: Dichoptic VR-based therapy did not restore perimetric field losses in ABI patients but reduced visual symptoms and may enhance functional adaptation of residual vision rather than structural recovery. The therapeutic response varied by etiology, favoring traumatic brain injury. Larger, longer trials integrating objective and subjective measures, including neuroimaging, are warranted.

## 1. Introduction

Acquired brain injury (ABI), encompassing traumatic brain injury (TBI), cerebrovascular accidents (CVAs), and brain tumors (BTs), remains one of the major causes of long-term disability worldwide. It has a profound impact on patients’ visual, cognitive, and motor functions [1,2].

People affected by ABI frequently present a broad spectrum of neurological sequelae, including emotional disturbances such as anxiety and depression, along with cognitive, behavioral, physical, and motor impairments. These limitations reduce autonomy and social participation, making multidisciplinary rehabilitation a long and essential process [1,2].

Around half of all patients with ABI experience some form of visual disturbance. Common manifestations include visual fatigue, blurred vision, and difficulty performing visually demanding tasks like reading. Visual field defects (e.g., hemianopia or quadrantanopia), loss of three-dimensional vision, photophobia, and oculomotor disorders such as strabismus or nystagmus are also frequent [3,4,5]. These problems are among the most disabling consequences of ABI, affecting mobility, reading ability, and daily functioning [6].

Stroke, in particular—the second leading cause of death and the third cause of disability globally—results in oculomotor dysfunction in up to 85% of cases. Despite this high prevalence, visual problems are often overlooked during post-stroke rehabilitation, even though nearly nine out of ten patients present some degree of visual impairment [7].

The visual pathway may be compromised at any level, from the retina to the visual cortex. Lesions of the optic nerve produce specific visual field defects reflecting the topographic arrangement of retinal fibers, whereas retrochiasmatic damage-involving the optic tract, lateral geniculate nucleus, optic radiations, or visual cortex-usually leads to homonymous hemianopia or quadrantanopia, often with macular sparing [8].

Neuroplasticity allows a certain degree of visual recovery during the first weeks or months after cortical injury. In later stages, visual rehabilitation programs can improve depth perception, eye movements, and spatial orientation [9,10]. The mechanisms underlying oculomotor dysfunctions after brain injury, however, are not fully understood; they often worsen under high cognitive load, indicating the involvement of attentional and executive cortical networks [11].

Oculomotor disorders are therefore common in ABI and can limit functional visual field performance. Within this framework, vision therapy (VT) aimed at improving oculomotor control may offer an effective strategy to optimize the remaining visual function rather than to regenerate damaged areas. Based on the principles of neuroplasticity, these programs seek to improve saccadic efficiency, reduce compensatory head movements, and enhance eye–hand coordination through structured, task-oriented exercises [12].

Despite the clinical relevance of these interventions, few studies have assessed their outcomes in ABI, and access to specific visual rehabilitation programs remains limited [3,4,5]. In recent years, virtual reality (VR)-based technologies have emerged as promising tools in this field, providing immersive, multisensory, and individualized environments. Devices such as Dicopt Home and Dicopt Pro deliver dichoptic and oculomotor training adapted to the patient’s residual visual field, potentially facilitating neuronal reorganization and functional recovery [13,14].

Immersive extended-reality (XR) systems encompass a continuum ranging from fully virtual environments (VR) to augmented (AR) and mixed reality (MR) solutions. Recent advances—such as holographic MR applied to procedural planning and postoperative follow-up in interventional cardiology [15]—illustrate how immersive environments can enhance diagnostic evaluation, therapy planning, and rehabilitation workflows across multiple medical specialties. Within this paradigm, dichoptic VR-based therapy constitutes one modality within a broader XR ecosystem, whose potential integration into neurorehabilitation is expanding rapidly.

Nevertheless, current evidence on the use of VR-based visual rehabilitation in ABI is still scarce. The present study aims to evaluate the effectiveness of a virtual reality-based vision therapy program (Dicopt Home) in individuals with acquired brain injury, combining objective visual field assessment with patient-reported visual symptom measures to explore both functional and perceptual outcomes.

## 2. Materials and Methods

### 2.1. Sample

The study included 52 participants with a confirmed diagnosis of acquired brain injury (ABI) (either stroke (CVA), brain tumor (BT), or traumatic brain injury (TBI)) who had achieved clinical stability for at least six months. Participants were recruited from the Neuro-ophthalmology Department at Gregorio Marañón Hospital and from several brain injury foundations and associations in the Madrid region, including the Spanish Brain Injury Federation (FEDACE), Daño Cerebral Invisible, and Freno al Ictus.

All procedures adhered to the tenets of the Declaration of Helsinki and were approved by the Ethics Committee of Hospital Clínico San Carlos (Madrid, Spain; reference 23/614-E, 18 October 2023). Participation was voluntary, and written informed consent was obtained from all participants.

Visual examinations were conducted at the Optometry and Vision Clinic of the Complutense University of Madrid (UCM). Data were recorded on standardized collection sheets and subsequently transferred to a computer database. For subjective symptom assessment, responses were gathered using digital forms created on the Google Forms platform.

Exclusion criteria included the absence of binocular vision, a time since brain injury of less than six months, or failure to provide signed informed consent. Participants were allocated to the therapy or control group using a strict alternation sequence (therapy–control–therapy–control), following the chronological order of recruitment. This procedure ensured balanced group sizes while maintaining a non-randomized allocation. Baseline comparability between groups was verified using Mann–Whitney U tests for continuous variables and chi-square tests for categorical variables.

Participants were recruited through two pathways: (1) referrals from the Neuro-ophthalmology Department of Hospital Gregorio Marañón, following routine clinical evaluation, and (2) voluntary participation through patient associations in the Madrid region, including FEDACE, Daño Cerebral Invisible, and Freno al Ictus. Recruitment was performed consecutively over the study period.

Inclusion criteria were confirmed diagnosis of ABI; clinical stability of at least six months post-injury; preserved binocular vision; ability to understand and follow task instructions; and absence of contraindications for the use of virtual reality devices. Exclusion criteria included ocular disorders unrelated to ABI (e.g., uncontrolled glaucoma, retinal disease), cognitive impairment interfering with VR task execution, photosensitivity, epilepsy, or incomplete participation in any study phase.

### 2.2. Experimental Protocol

At the first visit, participants completed the Brain Injury Vision Symptom Survey (BIVSS) questionnaire [15], a validated instrument for quantifying visual symptoms such as blur, double vision, photophobia, and reading difficulties in patients with acquired brain injury. A detailed anamnesis was then performed, followed by measurements of best-corrected visual acuity (VA), stereopsis, and ocular deviations using the cover test (CT). Finally, functional visual field (VF) testing was carried out using the Dicopt Pro virtual reality (VR) device (V-Vision S.L., Madrid, Spain), recording the following parameters:

- Percentage of detected stimuli (“seen/not seen”) per quadrant (%);
- Reaction time per quadrant (ms).

After baseline assessment, participants were assigned to two groups following a controlled parallel-group design with balanced allocation. Group 1 underwent six sessions of Dicopt Home vision therapy (VT), lasting 15 min each, while Group 2 served as the control group and received no treatment. Control participants were unable or unwilling to participate in therapy sessions. The dataset was divided according to an experimental design, with 27 participants assigned to the training group and 25 to the control group, ensuring balanced allocation.

The six VT sessions were organized into three game modules, each targeting different visual skills. Their order was randomized for each participant:

- Module 1: “Monster Game”—oculomotor training (2 sessions);
- Module 2: “Runner”—binocular vision training (2 sessions);
- Module 3: “Double Space”—vergence training (2 sessions).

**Figure 1:**
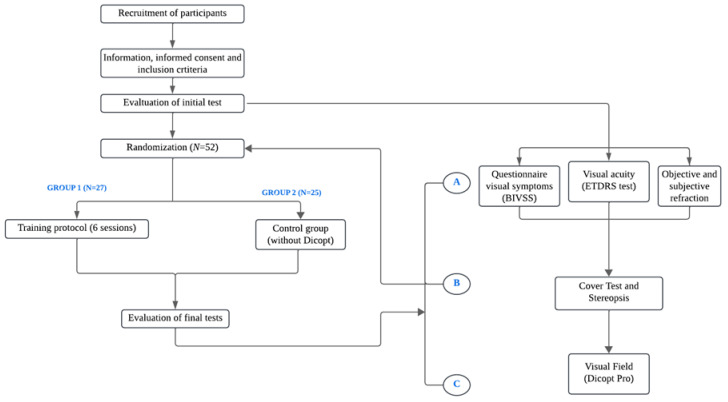
Simplified flowchart of the experimental protocol, showing participant recruitment, randomization, group allocation, intervention schedule, and pre–post visual assessments.

At the final visit, all baseline measurements were repeated in both groups to evaluate changes over time. A schematic flowchart of the experimental design is shown in Figure 1.

### 2.3. Materials

**Figure 2:**
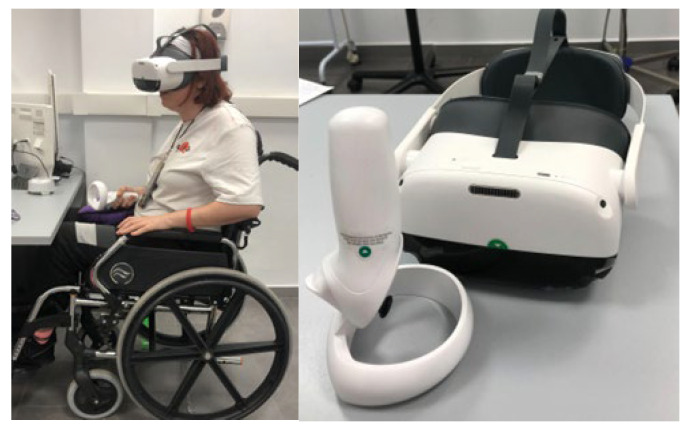
Functional visual field test using the Dicopt Pro virtual reality device.

- Brain Injury Vision Symptom Survey (BIVSS): Visual symptoms were assessed using the validated Brain Injury Vision Symptom Survey (BIVSS) questionnaire (Laukkanen et al., 2017 [16]), composed of 28 items addressing blur, double vision, glare, photophobia, eye discomfort, dryness, depth perception, peripheral awareness, and reading difficulties. Each item was rated according to symptom frequency, providing an overall score that reflects the perceived visual impact in patients with acquired brain injury.
- Visual Acuity (VA): Best-corrected VA was measured monocularly and binocularly using a standard ETDRS logMAR chart (Early Treatment Diabetic Retinopathy Study) at 4 m for distance and 40 cm for near vision. The smallest line correctly identified by the participant was recorded as the final VA score.
- Cover Test (CT): The CT was performed for both distance and near fixation to identify and quantify ocular misalignments using prism bars. Base-in (BI) prisms were applied for exodeviations, and base-out (BO) prisms for esodeviations. The fixation target corresponded to two lines below the VA of the worse eye.
- Stereopsis: Depth perception was evaluated at 40 cm using the Titmus stereo test with polarized glasses. The smallest measurable disparity in seconds of arc was recorded as the stereopsis threshold.
- Visual Field (VF): Binocular VF (120°) was evaluated using the Dicopt Pro virtual reality (VR) device, which integrates an eye-tracking system. After calibration, participants fixated on a central red cross and pressed a trigger each time they detected a peripheral stimulus. The test automatically paused if fixation was lost. A “seen/not seen” strategy was applied to assess the functional VF across quadrants, and results were automatically generated and exported for analysis (Figure 2).

- Dicopt Home Vision Therapy: The intervention was carried out using the Dicopt Home mobile application, a VR-based tool that allows patients to perform vision therapy (VT) either in a clinical or home-based setting. The system is designed to enhance binocular vision, accommodation, and oculomotor control by presenting distinct stimuli to each eye, thus promoting binocular integration.

**Figure 3:**
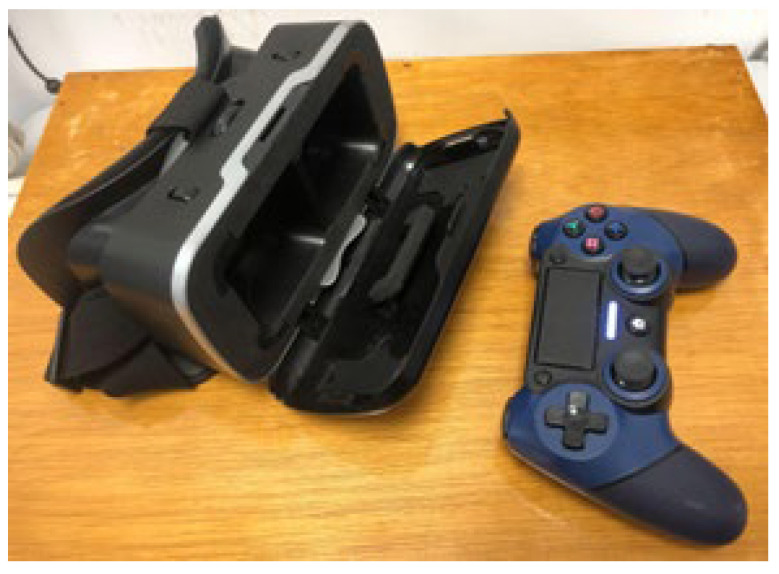
Dicopt Home virtual reality vision therapy setup.

A VR headset and joystick controller were required (Figure 3). The smartphone was placed inside the headset, allowing for adjustment of interpupillary distance (IPD) and focus. Participants interacted with various game-based exercises following on-screen instructions. All sessions were conducted under direct supervision by an optometrist to ensure proper task execution and patient safety. No adverse effects such as dizziness or nausea were reported.

The intervention was carried out using the Dicopt Home mobile application. For this study, we used Dicopt Home version 1.7.1. The software is developed by Innotec Visión Avanzada Xr, Sl (Madrid, Spain), operating under the commercial name V-Vision, and is certified with CE marking for medical use.

The dichoptic training module delivered by Dicopt Home follows a structured therapeutic design based on principles of binocular cooperation, neurosensory rebalancing, and oculomotor stimulation. The software implements a contrast-balancing algorithm that adjusts the relative luminance presented to each eye in real time, reducing the contrast delivered to the dominant eye and enhancing the input of the non-dominant eye. This mechanism aims to promote binocular integration by encouraging equal participation of both visual channels.

Task difficulty increased progressively across sessions by manipulating stimulus size, target complexity, relative interocular contrast, and temporal parameters such as presentation speed. This adaptive progression was designed to maintain engagement while facilitating gradual neurovisual recalibration. Each module required coordinated vergence, ocular tracking, or binocular alignment, ensuring sustained activation of visuomotor pathways. The therapeutic sequence and difficulty levels were predefined by the manufacturer and are consistent with protocols previously validated in dichoptic and oculomotor rehabilitation research.

**Figure 4:**
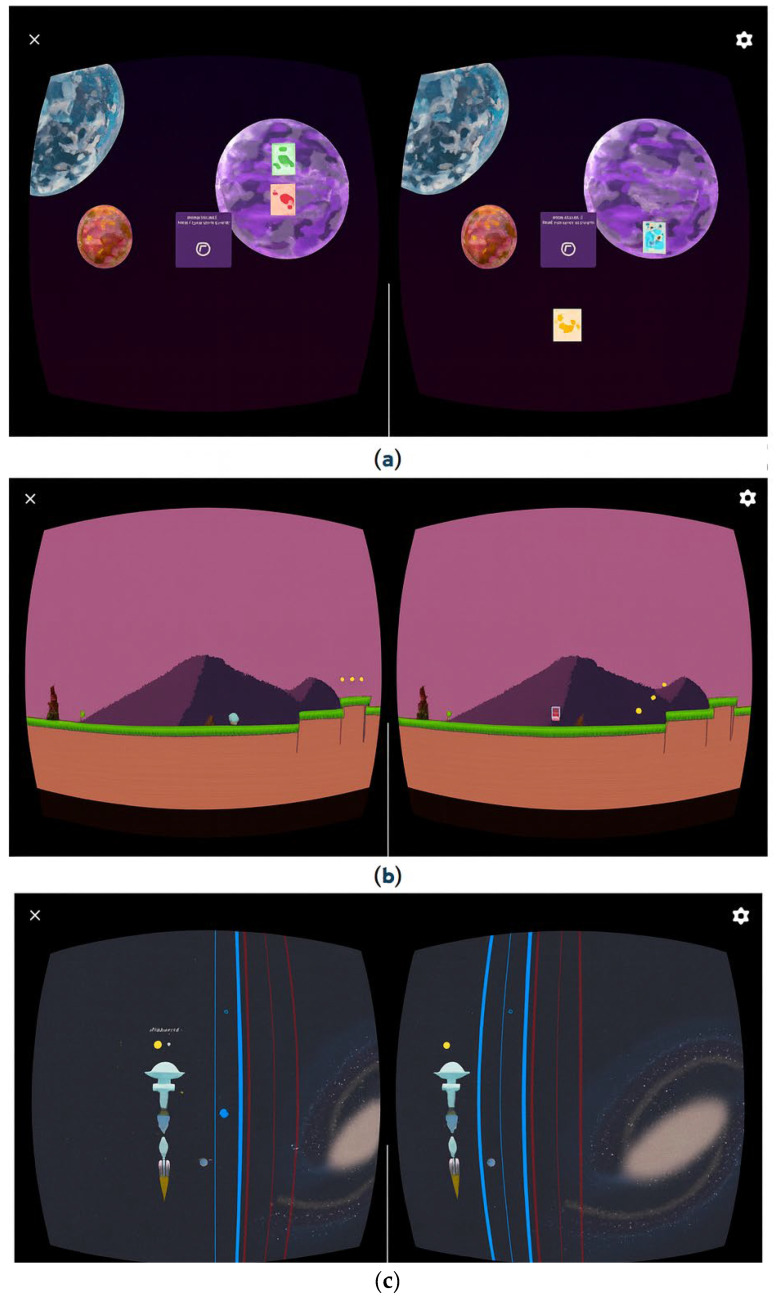
Dicopt Home game modules: (**a**) “Monster Game”, (**b**) “Runner”, and (**c**) “Double Space”.

The therapy included three interactive games:

- Monster Game: oculomotor training; participants aim and shoot only at the monster matching the central reference image (Figure 4a);
- Runner: binocular coordination training; the goal is to avoid obstacles and collect coins by pressing the “X” button to jump (Figure 4b);
- Double Space: vergence training; participants control two spaceships—blue (left joystick) and red (right joystick)—to avoid asteroids and collect stars of the corresponding color (Figure 4c).

### 2.4. Statistical Analysis

Data were analyzed using SPSS v30 (IBM Corp., Armonk, NY, USA). Descriptive and non-parametric statistics were applied, as the data did not meet normality assumptions (Shapiro–Wilk test, *n* < 50). Between-group comparisons were performed using the Mann–Whitney U test, and within-group comparisons using the Wilcoxon signed-rank test. A mixed ANOVA was conducted to examine differences in the top-right visual field quadrant. Although non-parametric tests were used for group comparisons, a mixed ANOVA was performed as an exploratory analysis to identify potential time × group interactions. Statistical significance was set at *p* < 0.05.

The main outcome variables were the percentage of detected visual stimuli and the reaction time (RT). Internal consistency was assessed using Cronbach’s alpha, yielding values of 0.847 for detection percentage and 0.937 for RT, indicating excellent reliability.

## 3. Results

A total of 52 participants were included (27 in the training group and 25 in the control group), with a balanced sex distribution (51.9% male and 48.1% female). The control group included 40% CVA, 40% BT, and 20% TBI cases, whereas the therapy group comprised 70.4% CVA, 14.8% BT, and 14.8% TBI, ensuring a balanced allocation of etiologies between groups. The main etiologies overall were cerebrovascular accident (CVA) in 55.8% of cases, brain tumor (BT) in 26.9%, and traumatic brain injury (TBI) in 17.3%. Descriptive characteristics by etiology are presented in Table 1.

At baseline, participants with BT were older and showed lower stereopsis (median 1976 arc sec, IQR 100–3552), while those with TBI presented greater near exodeviation (median −14 Δ, IQR −17 to −3). Participants with TBI also reported fewer visual symptoms in the BIVSS questionnaire (median 27, IQR 16.5–46.0) compared with those with BT (median 48.5, IQR 34.5–58.0).

After the intervention, a statistically significant increase was observed only in the top-right visual-field quadrant for the percentage of detected stimuli (median values increased from 79% [IQR 50.25–94.00] to 85% [IQR 53.00–94.00]; *p* < 0.001). No significant differences were found in the remaining quadrants or in reaction time (RT). Baseline comparisons between groups showed differences in the distance cover test (*p* = 0.047) and stereopsis (*p* = 0.003), so these variables were not included in the analysis of change. For visual-field outcomes, between-group differences in pre–post change were not significant (all *p* > 0.05), as summarized in Table 2.

Both groups showed a slight improvement in the top-right quadrant, with a median gain of 8% in the therapy group versus 2% in controls. Reaction time tended to increase in the control group and decrease in the therapy group, although with wide interquartile variability. A mixed ANOVA for the top-right quadrant revealed a main effect of time (*p* = 0.02), reflecting a general improvement across participants, but no group × time interaction (*p* = 0.069), indicating a similar trend of improvement in both groups.

When stratified by etiology, no significant between-group differences were observed in participants with CVA or BT in either the percentage of detected stimuli or reaction time, as shown in Table 3 and Table 4. In the TBI subgroup (*n* = 9), however, a significant difference was found in the top-left visual-field quadrant (*p* = 0.040), with a greater increase in the therapy group (median +5%, IQR 3–7.09) than in controls (median +2%, IQR −2–2). This finding should be interpreted with caution, given the small subgroup size and the heterogeneity of lesion locations among TBI participants. Reaction-time changes were not significant in any subgroup.

Regarding patient-reported outcomes, the BIVSS total score ranges from 0 to 100, with lower values indicating fewer visual symptoms. Scores decreased in the therapy group after intervention, indicating an improvement in perceived visual comfort. A significant within-group improvement was found only in the therapy group (median change −3 points, IQR −8 to 0; *p* = 0.015), while the control group remained stable (*p* = 0.202). The between-group comparison of BIVSS change was not significant (*p* = 0.144).

**Figure 5:**
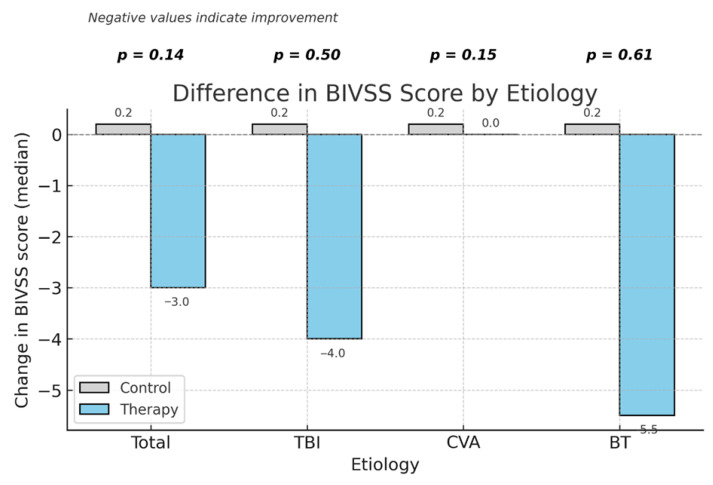
Median change in Brain Injury Vision Symptom Survey (BIVSS) scores between control and therapy groups by etiology. Negative values indicate improvement in visual symptoms. *p*-values correspond to the Mann–Whitney U test for intergroup comparison. TBI: traumatic brain injury, CVA: cerebrovascular accident, BT: brain tumor, BIVSS: Brain Injury Vision Symptom Survey.

When analyzed by etiology, no significant differences were found between groups; however, the therapy group showed a tendency toward lower post-intervention BIVSS scores—indicating reduced visual symptoms—in participants with TBI and BT, as illustrated in Figure 5. Overall, these findings suggest that the virtual reality-based vision therapy did not produce differential effects on visual field performance between groups, although individual improvements in visual perception were observed.

The satisfaction questionnaire was optional and was completed by 12 out of the 27 participants in the therapy group (44.4%), which is consistent with response rates commonly reported in rehabilitation studies. This reduced questionnaire assessed (1) the perceived effectiveness of the treatment in improving visual comfort and (2) overall satisfaction with the VR-based dichoptic therapy (Table 5).

For perceived effectiveness, 58.3% of participants reported being “satisfied” (score 4), 8.3% “very satisfied” (score 5), 16.7% “neutral” (score 3), and 16.7% “dissatisfied” (score 2). No participants selected “very dissatisfied” (score 1).

Overall satisfaction with the therapy was also high, with 41.7% reporting “very satisfied” (score 5), 33.3% “satisfied” (score 4), and 25% “dissatisfied” (score 2). No participants selected “neutral” or “very dissatisfied”.

These findings indicate good acceptability of the intervention and a generally positive perception of the VR-based rehabilitation program.

## 4. Discussion

The sample showed a balanced sex distribution and a median age of 51 years, representing a typical profile of adult patients with acquired brain injury (ABI). The binocular vision alterations observed, such as deviation in the cover test or reduced stereopsis, reflect the functional impact of ABI on the visual system, consistent with previous clinical reports [12,17].

In this study, most cases were associated with stroke, followed by cranial trauma (CT) and traumatic brain injury (TBI), a distribution that aligns with prior prevalence data [16]. This etiological diversity highlights the need to tailor therapeutic strategies to the underlying cause and clinical profile of each patient, as different lesion mechanisms may result in distinct visual patterns and rehabilitation responses. Lee YJ et al. [18] compared visual field defects in stroke and TBI patients, reporting that both groups exhibited alterations, although with different topographic characteristics. These findings underscore the importance of comprehensive visual evaluations and individualized rehabilitation protocols as part of the overall management of ABI patients.

In our study, no significant relationship was found between visual therapy (VT) and objective gains in the perimetric visual field (*p* > 0.05), except for a slight improvement in the top Right quadrant, where the experimental group showed a higher percentage increase. Reaction time measures also showed no relevant differences between groups. These results are consistent with numerous studies reporting limited or no perimetric recovery following rehabilitation [19,20,21]. Matteo et al. [19] emphasized the variability of rehabilitative effects on visual function, which may stem from heterogeneity in therapy protocols, treatment duration, and the targeted functional domains.

On the other hand, studies such as that by Smaakjær et al. [22] demonstrated that oculomotor-based VT improves fixation stability, reading efficiency, and visual attention, even without measurable expansion of the visual field. This supports the notion that functional improvement after ABI often results from compensatory mechanisms—such as enhanced scanning strategies or optimized saccadic control—rather than structural recovery of the damaged visual field. Similarly, Aimola et al. [20] and Hazelton et al. [21] reported significant functional improvements in daily activities following oculomotor training, despite the absence of perimetric changes, supporting the hypothesis of cortical reorganization or behavioral compensation rather than anatomical restoration.

The results from Mena-García et al. [23] also point in this direction: their compensatory saccadic training program for patients with hemianopia led to improvements in visual processing and functional independence, without measurable recovery of the visual field. Taken together, these findings—and our own results—reinforce the idea that visual rehabilitation in ABI enhances visual function and quality of life, even when structural restoration is not achieved.

Neuroimaging studies provide complementary evidence supporting these mechanisms. Nelles et al. [24] demonstrated that oculomotor training in post-stroke hemianopia induces cortical activation changes indicative of neuroplasticity. Kerkhoff et al. [25] and Willis et al. [26] reported increases in visual field size and perceptual performance after intensive training, suggesting that repeated stimulation may drive functional reorganization in visual areas. However, such benefits appear dependent on treatment duration and intensity—variables that, in our study (six sessions), may have been insufficient to produce measurable field changes.

Roth et al. [27] found that saccadic training improved reaction times and visual exploration efficiency, reinforcing the importance of extending the number or frequency of sessions to maximize functional outcomes. Future studies should therefore explore longer, more intensive interventions and include longitudinal follow-up to assess persistence of effects.

In our study, the intervention comprised only six 15 min sessions, which may be insufficient to induce robust neuroplastic changes. Previous research in oculomotor and perceptual rehabilitation suggests clear dose-dependent effects, with longer or adaptive training protocols producing more substantial and sustained improvements. Future studies should therefore consider extended or progressively adaptive therapy regimens to maximize functional gains.

An important aspect of our study concerns the influence of etiology on therapeutic response. Prior evidence indicates that patients with TBI may show greater neuroplastic potential and better oculomotor rehabilitation outcomes compared to those with stroke. Ciuffreda et al. [6,7] reported a 90% success rate in symptom improvement among TBI patients, while Kapoor et al. found significant gains in the developmental eye movement (DEM) test in the same population (*p* < 0.035). In line with these reports, our study also found a significant improvement in the top-left quadrant among TBI patients (*p* = 0.04), suggesting a higher capacity for functional recovery in this group.

Rowe et al. [17] and Rasdall et al. [28] similarly observed that TBI-related visual field defects tend to be more scattered and heterogeneous than those caused by stroke, possibly allowing greater compensatory adaptation. This variability in lesion type and extent likely accounts for some of the differences in VT response observed across studies.

Another contribution of this study lies in demonstrating the effectiveness of dichoptic visual therapy for reducing self-reported visual symptoms. Although no significant perimetric improvement was observed, the BIVSS scores showed a positive trend, consistent with previous studies using subjective symptom questionnaires [16,17,18,19]. However, few investigations have assessed the BIVSS in pre–post intervention designs or in conjunction with virtual reality (VR)-based therapies, underscoring its potential value as a complementary outcome measure in visual rehabilitation research.

Beyond perimetric performance, functional-vision metrics—such as reading speed, fixation stability, reaction-time variability, or ecological visuomotor tasks—may provide stronger correlations with perceived symptom improvement. Future iterations of the protocol should systematically incorporate these measures to obtain a more holistic evaluation of visual rehabilitation efficacy.

Although this study already incorporated structured subjective feedback through a satisfaction questionnaire completed by participants in the therapy group, additional qualitative approaches may further strengthen future protocols.

In addition to the structured satisfaction questionnaire collected in this study, future protocols could benefit from incorporating qualitative patient-reported experiences, such as semi-structured interviews or short narrative reports regarding comfort, perceived visual improvements, and usability of the VR system. Such qualitative data would provide richer contextual information that complements both the questionnaire-based outcomes and the objective clinical measures, helping to capture aspects of visual function, fatigue, motivation, or task engagement that may not be fully reflected in perimetric or functional-vision assessments.

The use of VR in neurovisual rehabilitation is a promising and expanding field. Vilageliu-Jordà et al. [13] highlighted the effectiveness of immersive VR in cognitive rehabilitation after ABI, noting improvements in motivation and treatment adherence. Likewise, Martino-Cinnera et al. [14] demonstrated that immersive VR combined with eye-tracking biofeedback can enhance spatial attention and visual exploration, suggesting that integrating VT with immersive environments may amplify neuroplastic and perceptual gains.

Recent developments in mixed reality (MR) further expand the potential of immersive technologies within clinical pathways. Holographic MR systems have demonstrated their utility for diagnostic visualization, procedural planning, and treatment monitoring in other medical domains, illustrating how extended-reality environments can enhance the integration of multimodal information and real-time feedback. Extrapolating these advances to neurovisual rehabilitation, dichoptic VR-based therapy may represent an initial step toward broader XR platforms capable of combining perceptual training, ecological task simulation, and longitudinal monitoring of functional outcomes.

Although no peer-reviewed clinical trials have yet evaluated the Dicopt system in individuals with ABI, previous studies of VR-based dichoptic therapy in amblyopia populations have reported significant improvements in stereoacuity and visual acuity [29,30]. This suggests that dichoptic stimulation in virtual reality environments may enhance binocular cooperation and oculomotor control, supporting the rationale for its use in ABI rehabilitation. Furthermore, the Dicopt system has obtained CE marking as a medical device in the European Union, reinforcing its technical and safety validation.

A key limitation of this study is the relatively small sample size, which may have reduced the statistical power to detect subtle functional differences between groups. Future studies, including larger and more homogeneous cohorts, are needed to confirm these preliminary findings.

Given the exploratory nature of the study and the modest sample size, the findings should be interpreted as preliminary. Nevertheless, they provide clinically meaningful trends that justify the development of larger, multicenter trials to more rigorously evaluate the efficacy of VR-based dichoptic interventions in ABI.

Another limitation is the absence of a sham or active control therapy, which may have influenced subjective improvements due to expectancy effects. Future studies should incorporate placebo-controlled or active-comparator designs to better isolate treatment-specific effects and minimize nonspecific therapeutic influences.

Overall, our findings align with current evidence indicating that visual rehabilitation in ABI patients (especially when incorporating dichoptic or immersive approaches) can reduce visual symptoms and improve functional outcomes, even if anatomical restoration of the visual field remains limited.

Future research should include larger samples, extended training programs, and multimodal assessment (e.g., fMRI, EEG) to better understand the neural mechanisms underlying recovery and to optimize therapeutic protocols.

## 5. Conclusions

Virtual reality-based dichoptic vision therapy did not produce significant changes in the functional visual field but improved self-reported visual symptoms. These results suggest an optimization of the intact visual function rather than structural recovery of the damaged area. The therapeutic response appeared to vary by etiology, with greater functional gains in TBI cases. Future studies should include longer training protocols and combined objective and subjective measures to comprehensively assess the efficacy of these interventions.

## Figures and Tables

**Table 1:** Descriptive characteristics of the total sample and by etiology group (TBI, CVA, and BT). Values are expressed as median and interquartile range (IQR).

		Total (*n* = 52)	TBI (*n* = 9)	CVA (*n* = 29)	BT (*n* = 14)
Age (years)		51.00 (38.25, 60.75)	50.00 (34.00–55.00)	51.00 (40.50–61.00)	54.50 (24.00–65.00)
VA (logMAR)		0.00 (−0.10, 0.10)	0.00 (−0.15, 0.08)	0.00 (−0.10, 0.01)	0.05 (0.00, 0.24)
Cover Test Far (Δ)		0.00 (−2.00, 0.00)	−2.00 (−9.00, 0.00)	0.00 (−1.50, 0.00)	0.00 (−2.25, 0.00)
Cover Test Near (Δ)		−3.00 (−8.00, 0.00)	−14.00 (−17.00, −3.00)	0.00 (−6.00, 0.00)	−4.00 (−6.00, 0.00)
Stereopsis (arc sec)		120.00 (60.0, 3552.0)	100.00 (40.00, 1976.0)	80.00 (45.00, 300.0)	1976.00 (100.00, 3552.0)
PPS—VF (%)	Top Right	79.00 (50.25, 94.00)	86.00 (47.50, 94.00)	77.00 (50.00, 89.50)	81.00 (47.75, 96.00)
	Bottom Right	88.00 (64.25, 98.00)	83.00 (48.50, 98.00)	90.00 (72.00, 98.00)	87.00 (48.75, 98.00)
	Top Left	75.50 (46.00, 92.00)	88.00 (53.50, 94.50)	71.00 (45.00, 91.00)	79.50 (40.75, 95.00)
	Bottom Left	91.50 (26.25, 100.00)	98.00 (61.50, 99.00)	88.00 (17.50, 100.00)	79.50 (44.75, 100.00)
RT—VF (ms)	Top Right	562.50 (511.50, 625.75)	548.00 (451.00, 575.00)	564.00 (506.50, 624.00)	582.50 (540.50, 636.50)
	Bottom Right	554.00 (487.50, 599.50)	523.00 (448.50, 575.50)	563.00 (493.00, 618.00)	556.00 (511.75, 594.25)
	Top Left	551.00 (494.75, 613.00)	523.00 (448.50, 575.50)	564.00 (503.50, 681.50)	561.00 (479.25, 625.00)
	Bottom Left	543.50 (491.25, 608.25)	503.00 (441.50, 587.50)	541.00 (491.50, 686.00)	557.50 (527.50, 612.75)
BIVSS (points)		35.50 (25.50, 54.75)	27.00 (16.50, 46.00)	32.00 (27.00, 57.00)	48.50 (34.50, 58.00)

VA: visual acuity, TBI: traumatic brain injury, CVA: cerebrovascular accident, BT: brain tumor, VF: visual field, PPS: percentage of points seen, RT: reaction time, ms: milliseconds.

**Table 2:** Comparison between control and therapy groups for baseline and pre–post change in visual field parameters. Values are expressed as median (IQR).

Variable (Unit)	Control Group (*n* = 25)	Therapy Group (*n* = 27)	Mann–Whitney U Test
Age (years)	50.0 (32.50, 62.50)	52.00 (42.00, 59.00)	0.63
Visual Acuity (logMAR)	0.00 (−0.10, 0.08)	0.00 (−0.14, 0.10)	0.14
Ocular deviation—Distance (Δ)	0.00 (−3.00, 0.00)	0.00 (−2.00, 0.00)	0.05
Ocular deviation—Near (Δ)	−4.00 (−6.00, 0.00)	0.00 (−8.00, 0.00)	0.59
Stereopsis (arc sec)	200.0 (100.00, 3552.00)	60.0 (40.00, 400.00)	0.003
Δ% Top Right VF	1.00 (−1.00, 3.00)	2.00 (−2.00, 8.00)	0.25
Δ% Lower Right VF	0.00 (−2.00, 1.00)	0.00 (−6.00, 8.00)	0.77
Δ% Top Left VF	2.00 (−1.50, 2.00)	2.00 (−4.00, 9.00)	0.31
Δ% Lower Left VF	0.00 (−2.00, 1.00)	0.00 (−6.00, 7.00)	0.99
ΔRT Top Right VF (ms)	1.00 (−2.00, 9.50)	−6.00 (−34.00, 32.00)	0.41
ΔRT Lower Right VF (ms)	0.00 (−6.00, 3.00)	−2.00 (−61.00, 15.00)	0.40
ΔRT Top Left VF (ms)	1.00 (−3.50, 5.50)	−3.00 (−58.00, 19.00)	0.43
ΔRT Lower Left VF (ms)	2.00 (−2.00, 21.00)	−8.00 (−50.00, 22.00)	0.10
ΔBIVSS (score)	0.00 (−2.00, 0.00)	−3.00 (−8.00, 0.00)	0.14

**Table 3:** Visual field changes by etiology: percentage of detected stimuli. Values are expressed as median (IQR).

Cause	Quadrant of the VF	Control Group	Therapy Group	Mann–Whitney U Test
TBI	Top Right	3.0 (3.0, 4.0)	5.0 (2.0, 22.0)	0.32
	Bottom Right	0.0 (−2.0, 0)	5.0 (−2.0, 29.5)	0.32
	Top Left	2.0 (−2.0, 2.0)	5.0 (3.0, 7.09)	0.04
	Bottom Left	0.0 (−1.0, 1.0)	2.0 (0.0, 8.5)	0.32
CVA	Top Right	1.5 (0.0, 5.0)	2.0 (−2.0, 8.0)	0.70
	Bottom Right	0.0 (−2.0, 2.0)	0.0 (−6.0, 8.0)	0.73
	Top Left	1.5 (−1.0, 2.0)	2.0 (−7.0, 9.0)	0.58
	Bottom Left	−0.5 (−1.0, 2.0)	−1.0 (−8.0, 7.0)	0.66
BT	Top Right	0.5 (−1.0, 2.0)	1.5 (−1.5, 8.0)	0.62
	Bottom Right	0.0 (−1.0, 1.0)	−3.0 (−5.0, 11.5)	0.22
	Top Left	1.5 (0.0, 2.0)	−2.5 (−5.0, 5.0)	0.35
	Bottom Left	−0.5 (−3.0, 1.0)	−1.0 (−4.0, 17.5)	1.00

TBI: traumatic brain injury, CVA: cerebrovascular accident, BT: brain tumor, VF: visual field.

**Table 4:** Visual field changes by etiology: reaction time (RT) per quadrant. Values are expressed as median (IQR).

Cause	Quadrant of the VF	Control Group	Therapy Group	Mann–Whitney U Test
TBI	Top Right	9.0 (5.0, 10.0)	−27.0 (−52.0, −5.5)	0.18
	Bottom Right	3.0 (−1.0, 18.0)	−31.5 (−75.0, −2.0)	0.07
	Top Left	2.0 (2.0, 7.0)	−22.0 (−68.5,27.5)	0.71
	Bottom Left	7.0 (2.0, 20.0)	−35.5 (−84.0,18.5)	0.39
CVA	Top Right	3.0 (−1.0, 15.0)	15.0 (−41.0, 51.0)	0.93
	Bottom Right	−1.0 (−7.0, 2.0)	1.0 (−53.0, 22.0)	0.82
	Top Left	1.5 (−3.0, 5.0)	−3.0 (−23.0, 19.0)	0.89
	Bottom Left	2.5 (−4.0, 22.0)	−19.0 (−50.0, 22.0)	0.16
BT	Top Right	−0.75 (−7.0, 1.0)	−25.5 (−31.0, 9.0)	0.18
	Bottom Right	−0.5 (−5.0, 6.0)	−26.5 (−63.5, 5.0)	0.52
	Top Left	0.0 (−4.0, 3.0)	−15.5 (−78.0, 3.5)	0.40
	Bottom Left	−0.5 (−1.0, 8.0)	2.5 (−24.5, 39.0)	0.94

TBI: traumatic brain injury, CVA: cerebrovascular accident, BT: brain tumor, VF: visual field.

**Table 5:** Scores ranged from 1 (very dissatisfied) to 5 (very satisfied). Percentages reflect the distribution of responses for the two questionnaire items administered to the therapy group (*n* = 12).

Score	Interpretation	Perceived Effectiveness	Overall Satisfaction
1	Very dissatisfied	0%	0%
2	Dissatisfied	16.7%	25%
3	Neutral	16.7%	0%
4	Satisfied	58.3%	33.3%
5	Very satisfied	8.3%	41.7%

## Data Availability

The data supporting the conclusions of this study are available upon request from the corresponding author. Due to ethical and privacy restrictions, the data are not publicly accessible.

## Abbreviations

The following abbreviations are used in this manuscript:

ABI	Acquired Brain Injury
BI	Base-In (prism)
BIVSS	Brain Injury Vision Symptom Survey
BO	Base-Out (prism)
BT	Brain Tumor
CT	Cover Test
CVA	Cerebrovascular Accident
DEM	Developmental Eye Movement Test
EEG	Electroencephalography
fMRI	Functional Magnetic Resonance Imaging
IPD	Interpupillary Distance
IQR	Interquartile Range
PPS	Percentage of Points Seen
RT	Reaction Time
TBI	Traumatic Brain Injury
VA	Visual Acuity
VF	Visual Field
VR	Virtual Reality
VT	Vision Therapy
